# Rotation, Strain, and Translation Sensors Performance Tests with Active Seismic Sources

**DOI:** 10.3390/s21010264

**Published:** 2021-01-03

**Authors:** Felix Bernauer, Kathrin Behnen, Joachim Wassermann, Sven Egdorf, Heiner Igel, Stefanie Donner, Klaus Stammler, Mathias Hoffmann, Pascal Edme, David Sollberger, Cédric Schmelzbach, Johan Robertsson, Patrick Paitz, Jonas Igel, Krystyna Smolinski, Andreas Fichtner, Yara Rossi, Gizem Izgi, Daniel Vollmer, Eva P. S. Eibl, Stefan Buske, Christian Veress, Frederic Guattari, Theo Laudat, Laurent Mattio, Olivie Sèbe, Serge Olivier, Charlie Lallemand, Basil Brunner, Anna T. Kurzych, Michał Dudek, Leszek R. Jaroszewicz, Jerzy K. Kowalski, Piotr A. Bońkowski, Piotr Bobra, Zbigniew Zembaty, Jiří Vackář, Jiří Málek, Johana Brokesova

**Affiliations:** 1 Department für Geo- und Umweltwissenschaften, Ludwig-Maximilians Universität München, 80333 München, Germany; Kathrin.Behnen@web.de (K.B.); j.wassermann@lmu.de (J.W.); egdorf@geophysik.uni-muenchen.de (S.E.); igel@geophysik.uni-muenchen.de (H.I.); 2 Federal Institute for Geosciences and Natural Resources, Stilleweg 2, 30655 Hannover, Germany; stefanie.donner@bgr.de (S.D.); Klaus.stammler@bgr.de (K.S.); Mathias.hoffmann@bgr.de (M.H.); 3 Department of Earth Sciences, ETH Zürich, Sonneggstrasse 5, 8092 Zürich, Switzerland; pascal.edme@erdw.ethz.ch (P.E.); david.sollberger@erdw.ethz.ch (D.S.); cedric.schmelzbach@erdw.ethz.ch (C.S.); johan.robertsson@erdw.ethz.ch (J.R.); patrick.paitz@erdw.ethz.ch (P.P.); jonas.igel@erdw.ethz.ch (J.I.); krystyna.smolinski@erdw.ethz.ch (K.S.); andreas.fichtner@erdw.ethz.ch (A.F.); 4 Department of Civil, Environmental and Geomatic Engineering, ETH Zürich, Stefano- Franscini-Platz 5, 8093 Zürich, Switzerland; rossiy@ethz.ch; 5 Institute of Geosciences, University of Potsdam, Karl-Liebknecht-Str. 24-25, 14476 Potsdam-Golm, Germany; gizem.izgi@uni-potsdam.de (G.I.); Daniel.Vollmer@geo.uni-potsdam.de (D.V.); eva.eibl@uni-potsdam.de (E.P.S.E.); 6 Institute of Geophysics and Geoinformatics, TU Bergakademie Freiberg, Gustav-Zeuner-Strasse 12, 09599 Freiberg, Germany; buske@geophysik.tu-freiberg.de; 7 Bayerisches Landesamt für Umwelt, Hans-Högn-Straße 12, 95030 Hof/Saale, Germany; christian.veress@lfu.bayern.de; 8 iXblue, 34 Rue de la Croix de Fer, 78100 Saint-Germain-en-Laye, France; frederic.guattari@ixblue.com (F.G.); theo.laudat@ixblue.com (T.L.); laurent.mattio@ixblue.com (L.M.); 9 Commissariat à L’Énergie Atomique et aux Énergie Alternatives (ou CEA), DAM, DIF, CEDEX, 91297 Arpajon, France; olivier.sebe@cea.fr (O.S.); serge.olivier@cea.fr (S.O.); charly.lallemand@cea.fr (C.L.); 10 Streckeisen GmbH, Daettlikonerstrasse 5, 8422 Pfungen, Switzerland; basil.brunner@streckeisen.biz; 11 Institute of Applied Physics, Military University of Technology, 2 gen. S. Kaliskiego Str., 00-908 Warsaw, Poland; anna.kurzych@wat.edu.pl (A.T.K.); michal.dudek@wat.edu.pl (M.D.); leszek.jaroszewicz@wat.edu.pl (L.R.J.); 12 Elproma Elektronika Ltd., 13 Szymanowskiego Str., 05-082 Łomianki, Poland; j.kowalski@elpromaelectronics.com; 13 Faculty of Civil Engineering and Architecture, Opole University of Technology, ul.Katowicka 48, 45-061 Opole, Poland; p.bonkowski@po.opole.pl (P.A.B.); p.bobra@po.opole.pl (P.B.); z.zembaty@po.edu.pl (Z.Z.); 14 Institute of Rock Structure and Mechanics, Czech Academy of Sciences, V Holešovičkách 41, 182 09 Prague, Czech Repulic; vackar@irsm.cas.cz (J.V.); malek@irsm.cas.cz (J.M.); 15 Department of Geophysics, Charles University, V Holešovičkách 2, 180 00 Prague, Czech Republic; johana.brokesova@mff.cuni.cz

**Keywords:** rotation sensors, strain sensors, seismology, instrumentation

## Abstract

Interest in measuring displacement gradients, such as rotation and strain, is growing in many areas of geophysical research. This results in an urgent demand for reliable and field-deployable instruments measuring these quantities. In order to further establish a high-quality standard for rotation and strain measurements in seismology, we organized a comparative sensor test experiment that took place in November 2019 at the Geophysical Observatory of the Ludwig-Maximilians University Munich in Fürstenfeldbruck, Germany. More than 24 different sensors, including three-component and single-component broadband rotational seismometers, six-component strong-motion sensors and Rotaphone systems, as well as the large ring laser gyroscopes ROMY and a Distributed Acoustic Sensing system, were involved in addition to 14 classical broadband seismometers and a 160 channel, 4.5 Hz geophone chain. The experiment consisted of two parts: during the first part, the sensors were co-located in a huddle test recording self-noise and signals from small, nearby explosions. In a second part, the sensors were distributed into the field in various array configurations recording seismic signals that were generated by small amounts of explosive and a Vibroseis truck. This paper presents details on the experimental setup and a first sensor performance comparison focusing on sensor self-noise, signal-to-noise ratios, and waveform similarities for the rotation rate sensors. Most of the sensors show a high level of coherency and waveform similarity within a narrow frequency range between 10 Hz and 20 Hz for recordings from a nearby explosion signal. Sensor as well as experiment design are critically accessed revealing the great need for reliable reference sensors.

## 1. Introduction

Rotation and strain, which are the antisymmetric and symmetric part of the wavefield displacement gradient tensor, respectively, are moving increasingly into the focus of geophysical research. One of the first attempts to derive seismic surface wave phase velocities from a combined observation of horizontal rotation and vertical translation by Schlüter [1] remained a theoretical consideration simply due to the lack of a suitable instrument measuring seismic ground rotations. The first observations of earthquake-induced rotational ground motions by large ring laser gyroscopes [2,3], as well as the observation of crustal coseismic deformation during the Landers earthquake sequence in 1992 by long baseline strainmeters [4], certainly count to the major milestones in seismic wavefield gradient observation. However, only very recent developments of portable seismic rotation and strain sensors made the direct observations of seismic wavefield gradients possible for a broad range of applications, such as volcanology [5,6,7], ocean bottom seismology [8,9,10], structural health monitoring [11,12,13], seismic exploration [14,15,16,17], microzonation in urban environments [18], and glaciology [19]. The most commonly used technologies for seismic ground rotation sensing are fiber-optic Sagnac interferometry [20], micro-electro mechanical systems [21], small-scale finite differencing within a rigid configuration of translation sensors [22], and liquid-based electrochemical transducers [23]. The technology of distributed acoustic sensing (DAS) makes the observation of seismically induced axial strain in temporary field experiments possible [24,25,26,27].

While, in classical seismology testing, standards exist for seismometers and accelerometers [28,29,30,31,32,33], only very few attempts have been made to introduce standardized testing procedures for seismic rotation and strain sensing instruments. Nigbor et al. [34] and Lee et al. [35] suggest performance test methodologies for strong motion rotational seismometers, addressing sensitivity and frequency response, clip level, self-noise, and resolution, as well as rotational and translational cross-axis sensitivity. Bernauer et al. [36] and Bernauer et al. [37] adapted a method that was originally proposed by Sleeman [38] for measuring the self-noise of traditional seismic sensors and digitizers to estimate self-noise levels of strong and weak motion rotational seismometers. The method uses three co-located sensors in order to minimize the influence of coherent signals to the self-noise estimation. The studies from Bernauer et al. [36] and Bernauer et al. [37] additionally investigate the scale factor variation with changes in the ambient magnetic field and ambient temperature while using a standard tilt table (CT-EW 01 by Lennartz Electronics). Schreiber et al. [11], Velikoseltsev et al. [39], Jaroszewicz et al. [40], and Kurzych et al. [41] access self-noise performance characteristics of fiber-optic gyroscopes measuring Allan deviation [42], a technique that is very well established in the field of inertial navigation, but relatively unknown in the seismological community.

Seismic rotation sensors can easily be calibrated absolutely for strong motion signals while using testing standards for inertial navigation instruments or standard tilt tables, as they are commonly used for absolute calibration of horizontal seismometer components [37]. However, these procedures are not suitable for relative calibration within a broad frequency range and weak motion signals. Manufacturers and users of classical seismometers measure the frequency response of their instruments while using calibration coils, shaking tables, or huddle tests involving very well known reference instruments. Calibration coils or similar approaches cannot be implemented in most seismic rotation sensors due to technical reasons. Shaking tables producing highly precise and reproducible rotational weak motion, especially with frequencies below 0.1 Hz, do not count to the standard equipment of seismological laboratories. Finally, for the relative calibration of rotation sensors in a huddle test, a reliable and well known reference sensor is missing. Another possibility for accessing the reliability of ground rotation measurements is the comparison with finite differences being obtained from classical seismometer arrays [43,44,45], which is also only valid for a limited frequency range.

In order to characterize the instrument response of fiber-optic cables used for distributed acoustic sensing, Wang et al. [46] and Lindsey et al. [47] co-located fiber-optic DAS-arrays with conventional seismometers and compared the direct strain measurement to the strain that was obtained by finite differencing of two seismometer waveforms. In these studies, the observations match well for signal periods from 10 s to 120 s. Paitz et al. [48] extended the frequency range for this kind of comparison and found a good match of observations for frequencies from 1/3000 Hz to 60 Hz.

As an important step towards improving data quality and reliability of rotation and strain sensing instruments, researchers from the Ludwig-Maximilians University of Munich (LMU), the Federal Institute for Geosciences and Natural Resources (BGR), the University of Potsdam and ETH Zürich organized a comparative sensor test experiment that took place in November 2019. We installed 24 rotational motion sensors and two fiber-optic cables for distributed acoustic sensing at the Geophysical Observatory of the LMU in Fürstenfeldbruck, Germany. Additionally, four permanent and 10 temporary seismic broadband stations and a 160 channel geophone chain recorded signals from explosions and Vibroseis sweeps. In this paper, we present the involved instruments as well as the setup of this first of its kind experiment, in detail. Subsequently, we show the first results concerning basic instrument performance characteristics, such as sensor self-noise levels and signal-to-noise ratios. We access waveform similarity for co-located recordings of an explosion by means of time-frequency analysis.

## 2. The Experiment

**Figure 1:**
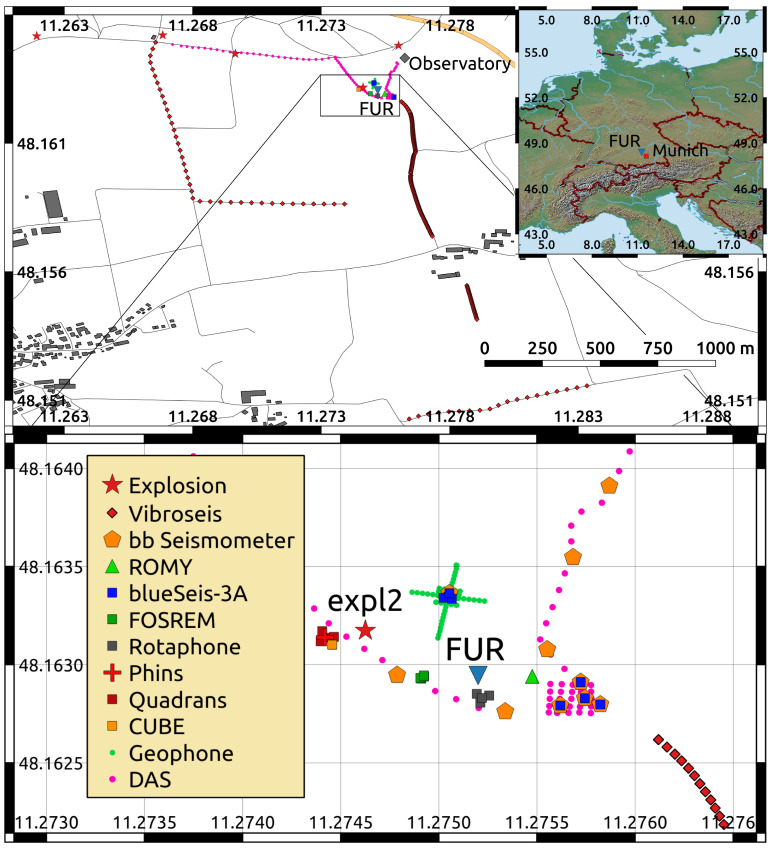
The Experiment was located at the Geophysical Observatory in Fürstenfeldbruck, approximately 25 km to the West of Munich, Germany. Five explosions were fired within a distance range from 50 m to 1.1 km from the instrument installations. The Vibroseis sweeps took place within a distance range of 20 m to 1.5 km to the instrument installations. For the huddle test, all of the rotation sensors were installed in the seismic bunker of the German Regional Seismic Network station FUR. For the active part of the experiment, the instruments were installed in different array configurations within a radius of around 60 m around FUR station.

The experiment took place approximately 25 km to the West of the city of Munich, Germany (Figure 1; for further information on the experiment the reader is invited to watch this video). The following sections describe the nominal performance characteristics of the tested instruments (for further details on the instruments and their working principles, the reader is referred to the references for the specific instrument), as well as the experimental setup, which consisted of two main parts: a huddle test and an active source part.

### 2.1. Instruments

In total, 41 different instruments measuring ground translation, rotation, and strain were tested in the experiment (see Figure 1). The group of rotation sensors is subdivided into four categories: (1) three-component (3C) as well as (2) single-component (1C) broadband rotational seismometers based on fiber-optic gyroscopes, (3) six-component (6C) strong-motion sensors combining fiber-optic gyroscopes and micro-electro mechanical systems, and (4) the six-component Rotaphone systems measuring translation and rotation by finite differencing. Additionally, we briefly describe the ROMY ringlaser gyroscope that is permanently installed at the test site, the distributed acoustic sensing system for strain observation, as well as the permanently and temporarily installed translation sensors.

#### 2.1.1. 3C Broadband Rotational Seismometers

The first commercially available fiber-optic gyroscope that is specifically designed for seismology is the blueSeis-3A by iXblue, France. The sensor is based on a closed-loop system, which means that its operating point is kept at the point of the highest sensitivity by an internal feedback loop [20]. With a nominal sensor self-noise level of 20 nrads${}^{-1}$Hz${}^{-1/2}$ in a frequency range from 0.01 Hz to 50 Hz and a minimum in Allan deviation smaller than 2 nrads${}^{-1}$ for integration times between 100 s and 500 s, it ranges among the most sensitive portable and field deployable rotational seismometers [37]. During the experiment, a total of eight blueSeis-3A sensors were tested (BS1, BS2, BGR, XB100, XB101, XB102, IXBLUE, and ISAE, owned by LMU, BGR, University of Potsdam, iXblue and ISAE-SUPAERO, Toulouse). For the active part of the experiment, four blueSeis-3A sensors (XB100, XB101, XB102, and IXBLUE) were installed in a triangular 3.4 m aperture array, co-located with a geophone grid (described later in the text). The other four blueSeis-3A sensors (BS1, BS2, BGR and ISAE) were installed together with four broadband seisometers in a triangular 6C-array with an aperture of 15 m co-located with a two-dimensional DAS grid (described later in the text).

#### 2.1.2. 1C Broadband Rotation Rate Sensors

A high-resolution rotational seismometer was jointly developed by Streckeisen GmbH, Switzerland, and Zurich University of Applied Science, Switzerland [49]. The one- component prototype, called FARO, which is based on the principle of an open-loop interferometric fiber-optic gyroscope, was installed during the experiment onto a seismic auxiliary platform within a distance of 7 m from the broadband station FUR. The key component of the instrument is a coil with a diameter of 70 cm and a very long fiber cable of 20 km length, leading to a nominal self-noise level of 5 nrads${}^{-1}$Hz${}^{-1/2}$ for a frequency range from 0.01 Hz to 50 Hz.

The Fibre-Optic System for Rotational Events & Phenomena Monitoring (FOSREM) is a single-component fiber-optic gyroscope that was constructed by the Military University of Technology, Poland, together with Elproma Elektronika Ltd., Poland. Two FOSREM sensors of type FOS3 (FOS3-1 and FOS3-2 in open-loop interferometric configuration) and two FOSREM sensors of type FOS5 (FOS5-1 and FOS5-2 in closed-loop interferometric configuration) were tested during the experiment. The minimum in Allan deviation for these sensors is approximately at 20 nrads${}^{-1}$ at integration times between 400 s and 500 s [41]. For the active part of the experiment, the FOSREM sensors were installed in a rectangular array with a maximum sensor distance of 1.9 m.

#### 2.1.3. 6C Strong Motion Sensors

Sensors that combine a triade of rotation rate sensors and a triade of accelerometers within one housing are commonly used as north-finding gyro compasses or inertial measurement units (IMUs) in navigation. With their compact design at the cost of a higher self-noise level, these instruments are expected to be highly useful in civil engineering applications, like seismic building monitoring. For the experiment, five of these sensors (one Phins (PHINS) and four Quadrans (QA181, QA296, QA381, and QA384) by iXblue, France) were roughly adapted to the needs of seismology in order to provide a continuously time-stamped six-channel data output. These sensors use a combination of three fiber-optic gyroscopes for rotation rate sensing and three Micro-Electro-Mechanical Systems (MEMS) for acceleration sensing. For the active part of the experiment, the Quadrans sensors were installed in a triangular-shaped array with an aperture of 5.8 m and the Phins sensor in the center.

Micro-Electro-Mechanical Systems (MEMS) are widely used for acceleration sensing in a huge variety of applications, which range from strong-motion or engineering seismology to inertial navigation. MEMS are relatively small (in the range of millimeters to a few centimeters) electronic components that combine logical circuits and mechanical structures on a single chip and combine advantages, such as low power consumption, compact design, and robustness. For the experiment, we attached three DC response MEMS accelerometers (Model 3711E1110G by PCB Piezotronics, Depew, USA) and three MEMS gyroscopes (one Horizon HZ1-100-100 by Systron Donner Inertial, Concord, USA, for vertical rotation rate sensing, and two Gladiator G150Z-100-100 by Gladiator Technologies, Snoqualmie, USA, for horizontal rotation rate sensing) to one rigid aluminum cube (subsequently called CUBE). The accelerometers have a nominal root-mean-square resolution of 2 mms${}^{-2}$ in a frequency range from 0.5 Hz to 100 Hz. The vertical gyroscope has a nominal resolution of 0.07 mrads${}^{-1}$ and a self-noise level of less than 0.04 mrads${}^{-1}$Hz${}^{-1/2}$ at 100 Hz, and the horizontal gyroscopes have a nominal resolution of 0.009 mrads${}^{-1}$ and a self-noise level of 0.017 mrads${}^{-1}$Hz${}^{-1/2}$. For the active part of the experiment, the MEMS-cube was installed at a distance of 5.2 m from the central instrument of the Quadrans-array.

#### 2.1.4. The Rotaphone Systems

The Rotaphone (developed jointly by the Institute of Rock Structure and Mechanics, Czech Academy of Sciences, and Charles University, Prague, Czech Republic) is a short-period mechanical sensor system, which records ground velocity and simultaneously determines the rotation rate by measuring the spatial velocity gradients at a point. Several pairs of parallel aligned geophones are attached to a rigid frame in order to make it possible to observe translational and rotational motions in three independent spatial directions. Rotaphones can resolve rotation rates that are as small as 10${}^{-8}$ rads${}^{-1}$ [50]. In the active part, the Rotaphone-CY systems were installed in a triangular array with an aperture of 5.2 m and one central station.

An important feature of Rotaphone measurements is a very precise calibration of the individual geophones, which consists of two parts: a laboratory pre-calibration that is performed by employing specialized equipment and the so-called in-situ calibration performed simultaneously with data processing and while taking the actual physical conditions as well as the geophones’ aging into account. Both types of calibration are necessary, especially for Rotaphone rotational records. All of the four Rotaphone-CY instruments, involved in the experiment, should have undergone a thorough laboratory calibration at the USGS Albuquerque Seismological Laboratory in April 2020. Unfortunately, it was not possible to perform that part until the time of writing this paper due to the world-wide covid-19 crisis (travel restrictions, etc.). A substitute form of the laboratory pre-calibration was performed utilizing facilities that are available in the Institute of Rock Structure and Mechanics in Prague. However, such a calibration does not cover the whole frequency range of the instruments; it is limited only up to 20 Hz, which is insufficient. Therefore, the Rotaphone-CY results should be considered to only be preliminary at this stage. They will be shown in detail in a separate paper.

#### 2.1.5. ROMY Ring Laser Gyroscope

The ROMY ring laser gyroscope that is hosted by the Geophysical Observatory in Fürstenfeldbruck is a unique instrument for measuring geodetic and seismic ground rotations [51,52]. Four independent, triangular shaped ring laser gyroscopes are arranged in a regular tetrahedron with a side length of 12 m and its tip pointing down. At the time of writing, the best performing ring can resolve vertical rotation rates in the range of 2 prads${}^{-1}$ at 100 s averaging time, in terms of Allan deviation [52].

#### 2.1.6. The Distributed Acoustic Sensing System

Distributed Acoustic Sensing (DAS) is a relatively new tool in seismology that exploits the phase shift of backscattered laser pulses that are traveling in a fiber-optic cable. The phase-shift change is quasi-linearly proportional to the change in axial strain induced on the cable by a propagating seismic wavefield [47,53]. Backscattered laser light traveling in a fiber optic cable returns to the interrogator unit at a time proportional to the distance at which the backscattering occurred. The so called gauge length, the distance along which interferometry is performed and the axial strain is determined, is often set around 10 m for seismic applications. The DAS method is capable of recording vibrations in a frequency range from mHz to kHz along the fiber. For the experiment, we deployed a DAS system consisting of an iDAS v2 interrogator and a cable of 1 km length containing two optical fibers (by SILIXA, Elstree, United Kingdom). Figure 1 shows the cable layout. One section of the fiber-optic cable formed a two-dimensional grid co-located with the 15 m aperture 6C blueSeis-3A array.

#### 2.1.7. Broadband Seismometers and Geophones

The Geophysical Observatory in Fürstenfeldbruck hosts four permanent broadband seismometer stations. The German Regional Seismic Network station FUR consists of a Streckeisen STS2.5 seismometer and a RefTek130 seismic recorder. The Bavarian Seismic Network station FFB1 consists of a Nanometrics Trillium 120 Posthole seismometer that is installed at a depth of approximately 80 m. The posthole stations FFB2 and FFB3 are equipped with Trillium Compact Posthole seismometers, also belonging to the Bavarian Seismic Network. The seismometers of the stations FFB1, FFB2, and FFB3 are connected to Nanometrics Centaur digitizers.

In addition to these permanent stations, ten temporary broadband seismometers were installed for the experiment along the fiber-optic cable (see Figure 1). Here, we used Nanometrics Trillium Compact and Nanometrics Horizon sensors in combination with either a Reftek130 or a Nanometrics Centaur recorder.

In a cross-shaped patch with an arm length of 30 m and a sensor spacing of 2 m, we installed 40 three-component and 40 vertical-component 4.5 Hz geophones (160 channels in total). In the central part of the cross, the geophones were arranged in a two-dimensional, rectangular grid of 10 m × 10 m.

### 2.2. The Huddle Test

**Figure 2:**
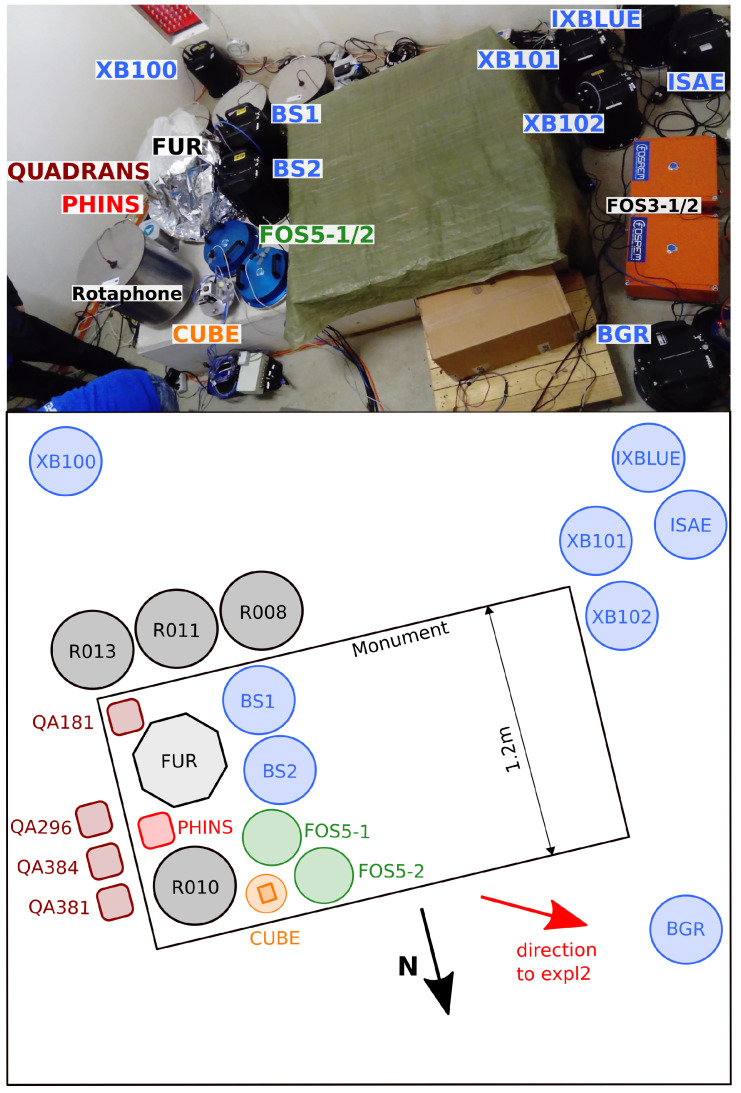
The huddle test took place in the seismic bunker of the German Regional Seismic Network station FUR. The lower panel shows a schematic overview of the huddle test site.

In a first part of the experiment, most of the tested sensors were co-located within the seismic vault of the German Regional Seismic Network station FUR (see Figure 1, label “FUR”, and Figure 2). Two blueSeis-3A sensors (BS1 and BS2), one Rotaphone-CY (R010), the MEMS-cube, the Phins, one Quadrans (QA181), and the two FOS5 sensors were directly installed onto the monument, which is seismically decoupled from the rest of the building. The rest of the rotation rate and 6C sensors were directly installed onto the ground of the seismic vault within a maximum distance between two sensors of 4.0 m (Figure 2). The FARO sensor was placed onto a seismic auxiliary platform within the same building, at a distance of 7.5 m to the main platform. The huddle test lasted from 19 November, 10:00 UTC to 20 November, 07:00 UTC, including the relatively quiet night time for self-noise estimation as well as two test explosions (150 g of explosive in a distance of 220 m to the huddle test site on 19 November, 10:26:04 UTC and 500 g of explosive in a distance of 52 m to the huddle test site on 19 November, 15:16:44 UTC) for comparisons of co-located recordings of signals that originate from a common source.

### 2.3. The Active Experiment

**Figure 3:**
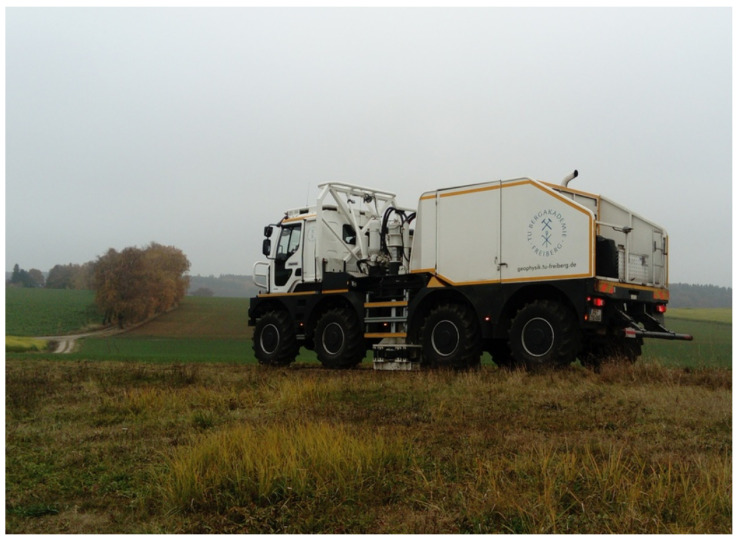
For the active part of the experiment a Vibroseis truck operated by the TU Bergakademie Freiberg (type: Thomas, VIB 3246) performed 480 sweeps.

In the second part of the experiment, the portable instruments were distributed on the observatory site (see Figure 1). In the outdoor installations during the active part of the experiment, most of the portable instruments were placed onto a rigid monument that consists of a 50 × 50 × 5 cm${}^{3}$ concrete plate. Instead of the concrete plate, a 30× 30 × 1.2 cm${}^{3}$ steel plate was used for installing the FOSREM sensors. The concrete and steel plates were dug around 50 cm deep into the ground and then leveled while using a thin layer of compacted sand between the plate and ground. Starting on 20 November, 11:00 UTC, until 21 November, 14:00 UTC, a Vibroseis truck (type Thomas, VIB 3246, operated by the TU Bergakademie Freiberg, see Thomas Constructeurs [54]) performed 480 sweeps at 160 different locations (Figure 3). The truck was operating at 30%, 50%, and 70% relative to a peak force output of 276 kN. The sweeps started at a frequency of 7 Hz and they ended at a frequency of 120 Hz. Each sweep lasted 15 s. The sweep sources were distributed over four different profiles (see Figure 1): two North–South profiles and two East–West profiles. The first North–South profile ranged from source–receiver distances of 75 m (measured from the central station FUR to the sweep point) up to 1000 m with azimuths between 114${}^{\circ }$ and 163${}^{\circ }$. For this relatively dense North–South Profile, the sweep point spacing was 5 m. The second North–South profile had a source spacing of 30 m and ranged from source-receiver distances of 681 m up to 698 m with azimuths between 230${}^{\circ }$ and 289${}^{\circ }$. The first East–West profile is directly connected to the second North–South profile with source receiver distances that ranged from 484 m up to 698 m and azimuths ranging from 191${}^{\circ }$ to 230${}^{\circ }$. The second East–West profile ranged from 154${}^{\circ }$ to 176${}^{\circ }$ azimuth in a source–receiver distance of approximately 1400 m with a source spacing of 30 m. Additionally, three times 1500 g of explosives were detonated at distances (azimuths) of 448 m (293${}^{\circ }$), 673 m (292${}^{\circ }$), and 1019 m (284${}^{\circ }$) on 20 November, 13:18:37 UTC, 13:46:16 UTC, and 14:17:44 UTC, respectively. The Bayrisches Landesamt für Umwelt (LfU) was responsible for the explosions.

## 3. Results and Discussion

In the following, we first present the results from the huddle test concerning basic instrument performance characteristics of rotation rate sensors. We analyze the instrument self-noise levels, characterize waveform similarity from recordings from the huddle test explosion “expl2” that happened at 15:16:43 UTC on 19 November 2019 (see Figure 1), study signal-to-noise ratios within the frequency range of highest coherency, and finally, compare selected recordings to a median reference waveform.

It is important to note that for the following waveform comparisons, the recorded traces were shifted in time. The applied time shifts are the result of maximizing the Pearson cross-correlation coefficient [55] between the vertical translational acceleration that was recorded by the reference station FUR and the transverse rotation rate that are expected to be in phase (see e.g., [56]). Table 1 summarizes the maximum Pearson cross-correlation coefficients and the applied time shifts, as well as the time source, synchronisation method, sampling rate, and location within the seismic bunker of each instrument analyzed in the following. Different reference clocks and time synchronisation methods that were used in the various recording units, as well as causal low-pass filtering prior to digital sampling decimation and data archiving, give reasons for these time shifts. blueSeis-3A sensors retrieve the absolute time from Global Navigation Satellite System (GNSS) and continuously synchronize the recorder time to a highly accurate pulse per second (PPS) signal. This leads to relatively low time shifts between 5 ms and 30 ms (see Table 1). The differences in time shifts between the blueSeis-3A sensors come from different low-pass filters that were implemented in different firmware versions prior to 200 Hz decimation. The prototype sensor IXBLUE uses a simple moving average filter prior to 200 Hz sampling rate decimation, theoretically introducing a latency of 2.5 ms. The prototype sensor BS1 additionally uses a Butterworth low-pass filter with non-linear phase response leading to a theoretical latency between 2.5 ms and 14 ms. All other bluesSeis-3A sensors use an anti-alias filter cascade introducing a latency of 30 ms. Within the uncertainty of one sample duration (5 ms), the values that are reported in Table 1 agree well with the theoretically calculated latency values for the different filter types that were provided by the manufacturer. However, this time shift is still not acceptable, especially for the analysis of frequencies above 10 Hz, where such a time shift exceeds 10% of a single period. Other time keeping methods, like synchronising the recorder time only once a day to a PPS signal (as it is done in the Phins and Quadrans sensors) or taking the absolute time stamp from Network Time Protocol (NTP, as it is done in the CUBE and FOSREM sensors), lead to even larger time shifts of almost 0.9 s. The Rotatphone-CY systems use standard seismic recorders that are synchronized to a GNSS/PPS clock. The observed time shift of 200 ms comes from causal low-pass filtering during post-processing, according to the manufacturer.

### 3.1. Instrument Self-Noise

**Figure 4:**
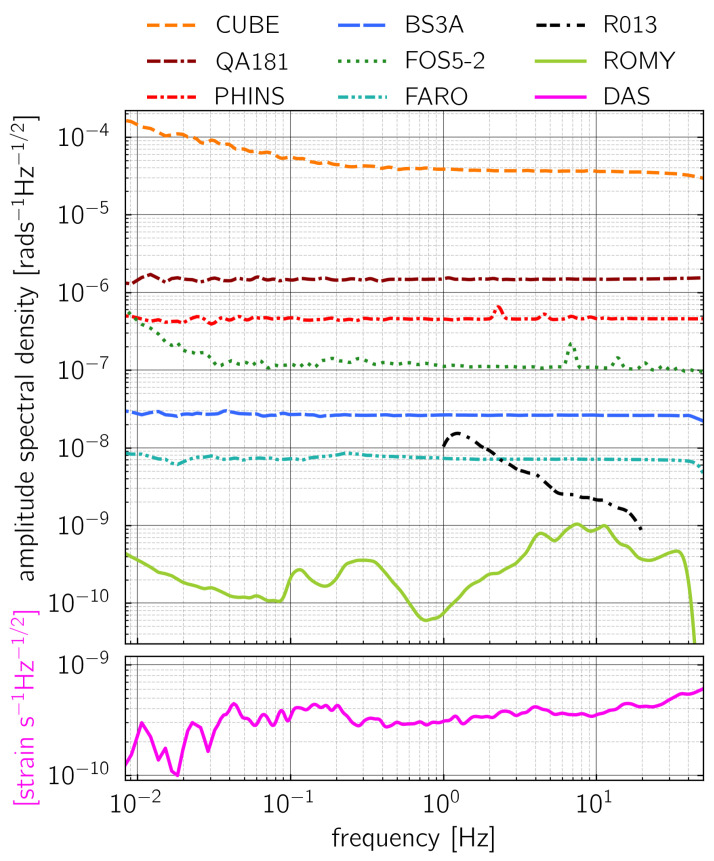
Amplitude spectral densities from night time recordings of vertical rotation rate during the huddle test on 19 November 2019. The spectrum from the ROMY ringlaser recording represents the seismic background noise. The spectra from the other rotation rate sensors represent the instrument self-noise of the tested rotational motion sensors. The amplitude spectrum of a parallel recording of the axial strain rate at the DAS measurement point closest to the huddle test site pointing to 300${}^{\circ }$ with respect to North is shown in the lower panel.

One of the first steps in characterizing instrument performance is to analyze the instrument self-noise level. The instrument self-noise is the output of the sensor, when the sensor is at rest and no input motion is present. It determines the lower limit of the sensor resolution. Estimating the instrument self-noise requires a recording from a seismically quiet time span with a background level of ground motion lower than the sensor self-noise level. This assumption can be verified, e.g., with a parallel and co-located recording from a reliable reference sensor. One excellent opportunity for analyzing the output of the sensors at rest is the night time recording during the huddle test. During this period, the rotation sensors were co-located within the seismic vault of the station FUR (see Figure 1 and Figure 2). Figure 4 displays the amplitude spectral densities calculated from parallel recordings of vertical rotation rate starting at 20:00:00 UTC on 19 November and lasting four hours. The amplitude spectral density of the ROMY ring laser recording shows that no input ground motion is large enough to exceed the self-noise levels of the other rotation rate sensors occurred during that time span within a frequency range from 0.01 Hz to 50 Hz. The fiber optic gyroscopes FARO, blueSeis-3A, Quadrans, and Phins show the typical flat self-noise spectra, which is indicating white noise in the frequency range from 0.01 Hz to 50 Hz. The self-noise level of the FOSREM sensor increases at frequencies that are below 0.03 Hz, which is connected to the use of standard single mode optical fiber in combination with depolarized light. In the frequency range from 0.01 Hz to 50 Hz, the FARO shows the lowest sensor self-noise level between 6 nrads${}^{-1}$Hz${}^{-1/2}$ and 8 nrads${}^{-1}$Hz${}^{-1/2}$, among the portable broadband rotation rate sensors. The Rotaphone-CY has a self-noise level of 16 nrads${}^{-1}$Hz${}^{-1/2}$ at 1 Hz decreasing to 1 nrads${}^{-1}$Hz${}^{-1/2}$ at 20 Hz. With the highest self-noise level of 40 rads${}^{-1}$Hz${}^{-1/2}$ for frequencies higher than 0.3 Hz, the MEMS based CUBE sensor is only useful for strong motion recording. In addition, we show the amplitude spectral density calculated from a DAS recording at the point closest to the huddle test site. The record was analyzed in the same time span as for the rotation rate sensors. The amplitude spectral density level for this relatively quiet time period is below 0.5 nanostrain s${}^{-1}$Hz${}^{-1/2}$.

### 3.2. Waveform Similarity

During the huddle test on 19 November at 15:16:45 UTC, 500g of explosive were detonated at a distance of 52 m from the huddle test site. This explosion opens a unique opportunity to compare co-located rotational motion recordings in the time as well as in the frequency domain. We explicitly exclude the recordings from ROMY and FARO from the subsequent comparison because these sensors were installed in slightly different locations when compared to the rest of the rotational motion sensors. Additionally, the CUBE sensor is excluded from the comparison because its signal-to-noise ratio for frequencies below 100 Hz and for the vertical component was too low for a reliable comparison to other sensors.

**Figure 5:**
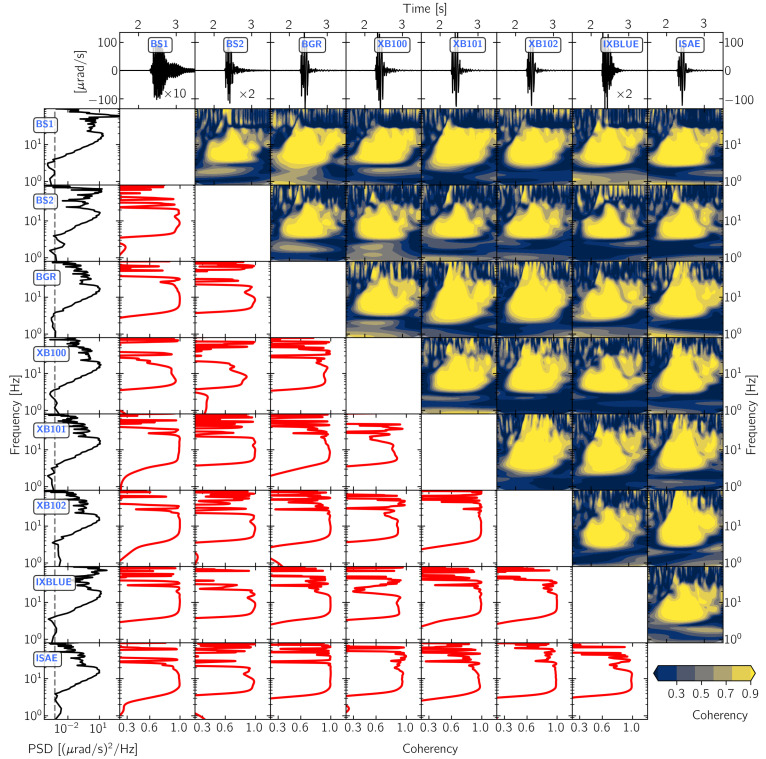
Waveform coherency for pairs of 3C rotational seismometers. The North component recording from explosion “expl2” on 19 November 2019 at 15:16:45 UTC (see Figure 1) is shown. The first row displays the raw waveforms and the first column displays the corresponding power spectral densities with the dashed line indicating the sensor self-noise level. The red graphs in the lower diagonal half represent Fourier-domain coherency spectra. The upper diagonal half shows time resolved wavelet coherency spectrograms.

**Figure 6:**
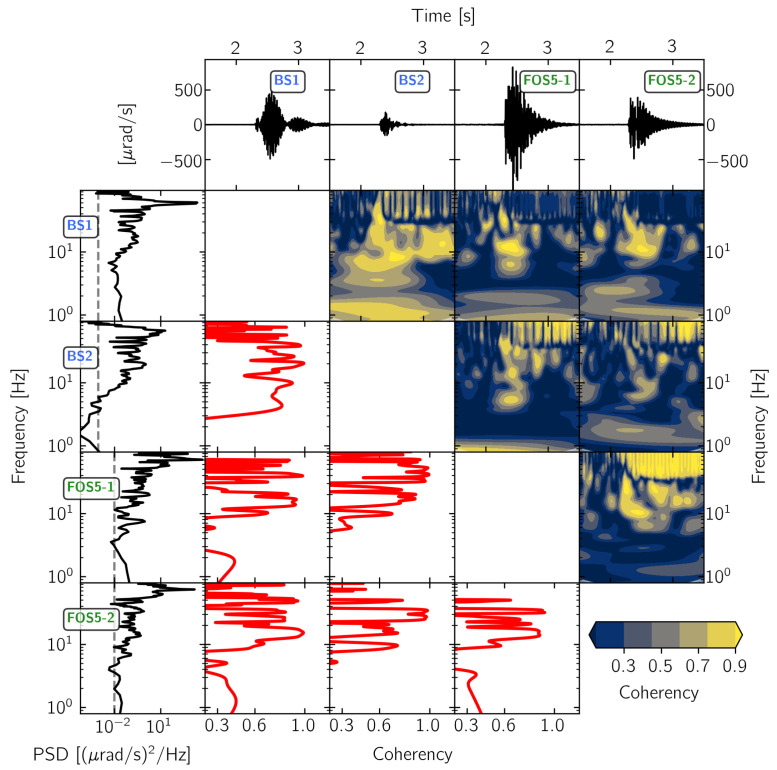
Waveform coherency for pairs of 1C vertical rotation rate sensors. The vertical component recording from explosion “expl2” on 19 November 2019 at 15:16:45 UTC (see Figure 1) is shown. For more details see Figure 5.

**Figure 7:**
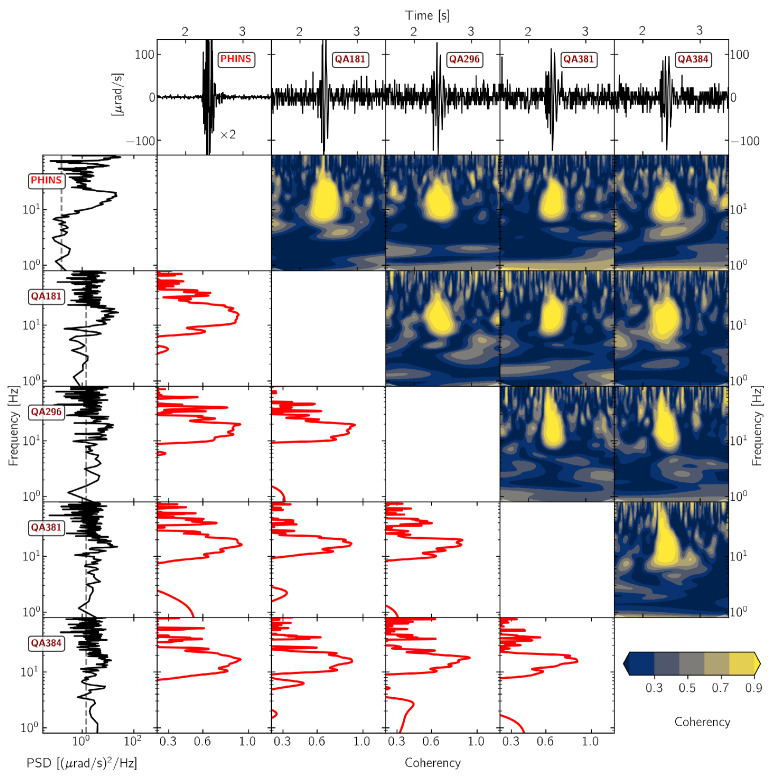
Waveform coherency for pairs of 6C strong motion sensor. The North component recording from explosion “expl2” on 19 November 2019 at 15:16:45 UTC (see Figure 1) is shown. For more details see Figure 5.

**Figure 8:**
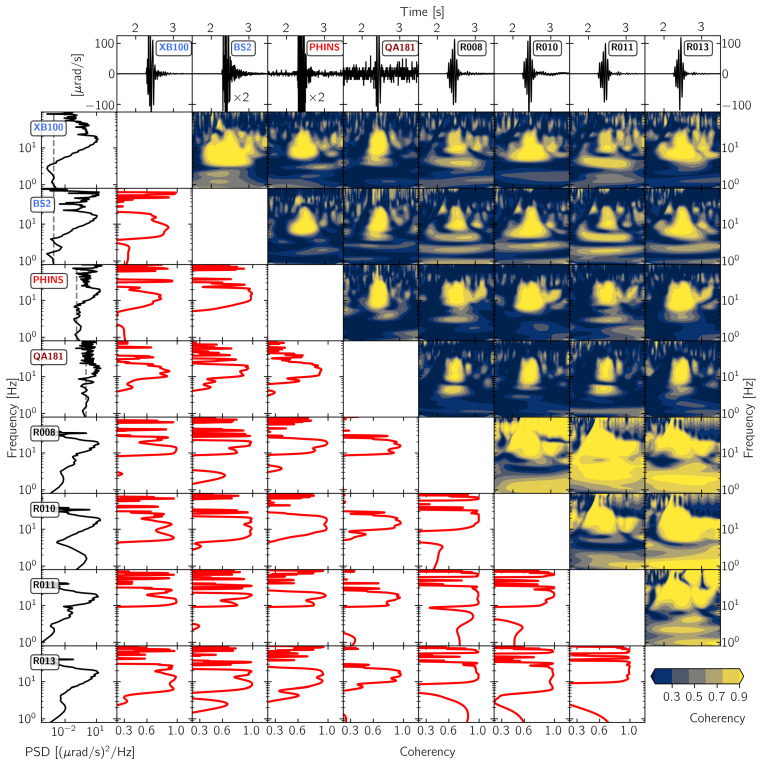
Waveform coherency for pairs of Rotaphone-CY systems as well as two blueSeis-3A rotation rate sensors and two strong motion fiber optic gyroscopes (Phins and Quadrans). The North component recording from explosion “expl2” on 19 November 2019 at 15:16:45 UTC (see Figure 1) is shown. For more details, see Figure 5.

Figure 5, Figure 6, Figure 7 and Figure 8 show the results of this time- and frequency-domain comparison for 3C and 1C broadband rotational seismometers, 6C strong-motion sensors, and Rotaphone-CY systems. The figures are all organized in the same way: the top row shows the raw waveforms that were recorded by the North component of the corresponding sensor. In case of single component sensors, we show the vertical rotation rate recording. For the sensors BS1, BS2, IXBLUE, and PHINS, we applied a scale factor that is displayed within the corresponding panel, in order to show the same y-axis range for all of the panels. The first column shows the corresponding power-spectral densities with the dashed line indicating the sensor self-noise level. The lower diagonal half shows the frequency-domain coherency between each pair of sensors. This representation is strong in analyzing signal similarity with respect to frequency but does not provide information on when in time the coherent signals occur. Therefore, the upper diagonal half shows time-frequency coherency analysis between each pair of sensor using analytic Morlet wavelets as basis functions [57]. This time-frequency analysis is capable of uncovering time resolved locally phase locked behavior between two time series [58]. However, this approach is a trade off between low temporal resolution for low frequencies and high temporal resolution for high frequencies.

#### 3.2.1. 3C Broadband Rotational Seismometers

The frequency domain analysis presented in Figure 5 shows that the signal power exceeds the self-noise levels of 3C broadband rotation seismometers for frequencies above 4 Hz reaching the highest power at 20 Hz. Accordingly, the recorded signals show very high coherency of almost 1 from 4 Hz to 20 Hz. For the sensors BS1, BS2, and IXBLUE, the power spectra in Figure 5 show high power for frequencies above 60 Hz. For BS1, this high frequency part of the signal clearly dominates the waveform and it shows low coherency (partly below 0.3) with signals being recorded by the other sensors. Additionally, the sensors BS2 and IXBLUE show dominant high frequency content (above 60 Hz), but not to the extent observed for BS1. Among the 3C broadband rotational seismometers, only BS1 and BS2 were installed on top of the decoupled monument (see Figure 2), which explains the undamped high frequency content in their recordings. The sensors BS1 and IXBLUE are prototype versions that were running without appropriate anti-alias filtering. This explains the high frequency content in their recordings and, especially for BS1, the poor coherency with other sensors.

The time-frequency analysis reveals high signal similarities for all sensor combinations in a frequency range between 4 Hz and 20 Hz for the time after the signal onset at approximately 2.2 s. The high coherency for frequencies that are below 10 Hz prior to the signal onset is an artifact coming from the low temporal resolution of wavelet time-frequency analysis at lower frequencies. The sensors BGR, XB101, XB102, and ISEA show high coherency values of almost 1 also for frequencies above 20 Hz after the signal onset. These sensors were installed close to each other on the floor of the building with their North component approximately in-line with the wave front generated by the explosion, as seen in Figure 2.

#### 3.2.2. 1C Broadband Rotation Rate Sensors

For the analysis of signal similarities between 1C vertical rotation sensors, we include the vertical component recordings of BS1 and BS2. Figure 6, again, reveals that sensors mounted on top of the monument see relatively high power at frequencies above 60 Hz, also in the vertical component. The sensors FOS5-1 and FOS5-2 show high coherency with BS1 in a narrow frequency band between 10 Hz and 20 Hz, while, in general, signal power exceeds the sensor self-noise levels for frequencies higher than 5 Hz. Looking at the time resolved wavelet coherency spectra, this coherent part of the signal appears for approximately 0.5 s right after signal onset. The vertical component recordings of BS1 and BS2 show higher coherency for a larger part of the signal in frequency as well as in time domain, however, only rarely exceeding a value of 0.7. Taking into account that these four sensors were mounted on top of the seismic monument within a maximum distance of 1 m, we would have expected much higher coherency and attribute this inconsistency to the prototype status of BS1, FOS5-1, and FOS5-2.

#### 3.2.3. Strong Motion Sensors

For the strong motion sensors, the signal power exceeds sensor self-noise levels for frequencies above 7 Hz in the case of the PHINS sensor and for frequencies above 10 Hz in the case of the Quadrans sensors (see Figure 7), reaching a maximum at 20 Hz. All of the sensors show high coherency values above 0.9 in a narrow frequency band between 10 Hz and 20 Hz, which coincides with the time span of approximately 0.5 s after the signal onset. The sensor PHINS, installed on top of the monument, recorded higher power in the frequency range above 60 Hz when compared to the other strong motion instruments.

#### 3.2.4. Rotaphone Systems

In this section, we compare Rotaphone-CY recordings to each other and additionally include recordings from XB100, BS2, PHINS, and QA181 to the comparison. XB100 was installed close to the three Rotaphone-CY systems on the floor of the building and BS2, PHINS, and QA181 were installed on top of the monument, together with the Rotaphone-CY system R010 (see Figure 2). The comparison between Rotaphone-CY systems in Figure 8 reveals high coherency values above 0.9 for a narrow frequency band between 10 Hz and 20 Hz between all Rotaphone-CY systems. High coherency between 5 Hz and 10 Hz can be observed between R010 and R013. The time-frequency analysis relates this region of high coherency to a wide time span around the main signal. As the only Rotaphone-CY system installed on top of the monument, R010 shows high coherency with BS2, PHINS and QA181, reaching values above 0.9 for frequencies between 5 Hz and 20 Hz. Note the high coherency of almost 1 for frequencies from 5 Hz to 20 Hz between R013, XB100. For a narrow time window of about 0.5 s, after the signal onset, all of the sensor combinations show high coherency above 0.9 in the frequency band between 10 Hz and 20 Hz.

### 3.3. Signal-To-Noise Ratio

**Figure 9:**
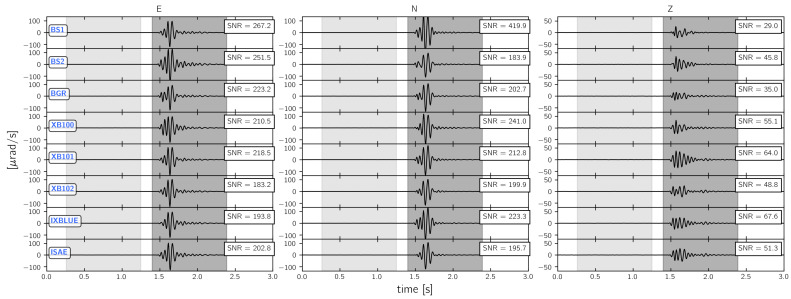
30 Hz low pass filtered waveforms from the explosion “expl2” (see Figure 1) recorded by 3C rotational seismometers. The light and dark shaded areas represent the parts of the time series from which noise root-mean-square (RMS) and signal RMS values are calculated, respectively. The obtained signal-to-noise ratios (SNR) are given for each instrument and each axis in the black box.

**Figure 10:**
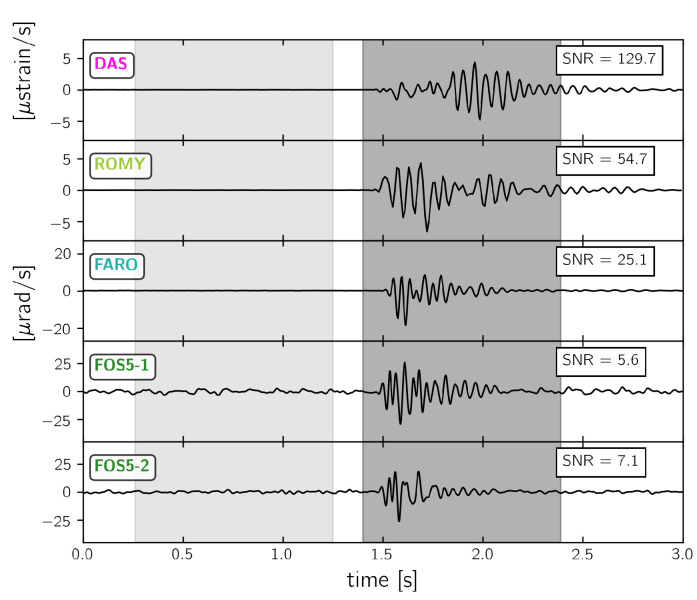
30 Hz low pass filtered waveforms from “expl2” (see Figure 1) recorded by 1C vertical rotation rate sensors and the DAS system at the point closest to the huddle test site. See Figure 9 for more details.

**Figure 11:**
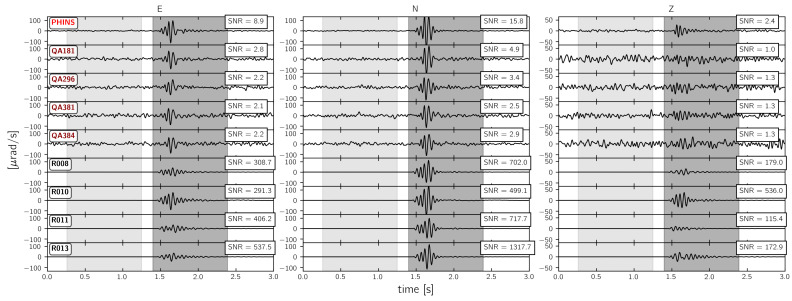
30 Hz low pass filtered waveforms from “expl2” (see Figure 1) recorded by 6C strong motion sensors as well as the Rotaphone-CY systems. See Figure 9 for more details.

As shown in the previous section, the strongest signal similarities appear for frequencies below 30 Hz. Therefore, the following signal-to-noise ratio analysis focuses on 30 Hz low-pass filtered waveforms. Figure 9, Figure 10 and Figure 11 display the recorded waveforms and show the corresponding signal-to-noise ratios. The signal-to-noise ratios were calculated from root-mean-square values of 1.0 s long time spans before and after the signal onset (grey shaded areas in Figure 9, Figure 10 and Figure 11).

#### 3.3.1. 3C Broadband Rotational Seismometers

Figure 9 shows the signal recorded by the portable 3C rotational seismometers. Signal-to-noise ratios range from 267 to 183 for the East component, from 241 to 184 for the North component with an exceptionally high value of 420 for BS1 and from 68 to 29 for the vertical component. The lower signal-to-noise ratio for the vertical component is related to the fact that vertical rotations are poorly excited by an explosive source. The six 3C rotational seismometers installed on the floor show signal-to-noise ratios varying by ±10% for the East and the North component. BS1 and BS2 mounted on top of the monument show slightly larger signal-to-noise ratios for the East component, while the signal-to-noise ratio for the North component of BS1 is extremely large. This behavior can be related to the prototype status of BS1 involving a non appropriate anti-alias filter and to the overall lower self-noise level of BS1 as it was analyzed by Izgi et al. [59].

#### 3.3.2. 1C Rotation Rate Sensors and DAS

Figure 10 shows waveforms and signal-to-noise ratios from the explosion as recorded by 1C vertical rotation sensors as well as by the DAS system. Among the rotation sensors, ROMY and FARO show the largest signal-to-noise ratios of 55 and 25, respectively, followed by FOS5-2 and FOS5-1. This behavior clearly reflects the sensor self-noise levels, which is the lowest for ROMY and higher for FARO and the FOSREM sensors (see Figure 4). The signal-to-noise ratio of the DAS system reaches a value of 130.

#### 3.3.3. Strong Motion Sensors and Rotaphone Systems

Figure 11 shows the waveforms and signal-to-noise ratios from the explosion signal that was recorded by the 6C strong motion sensors. The three Quadrans sensors installed on the floor (QA296, QA381, and QA384) show signal-to-noise ratios between 2.1 and 3.4 for the North and East component and 1.3 for the vertical component. QA181, mounted on top of the monument shows larger signal-to-noise ratios on the East and the North component and a smaller signal-to-noise ratio on the vertical component. Because of its lower self-noise level, the PHINS shows larger signal-to-noise ratios of 8.9 and 15.8 for the East and North component, respectively, and of 2.4 for the vertical component.

The Rotaphone-CY systems recorded the explosion signal with signal-to-noise ratios that ranged from 291 to 538 for the East component, from 500 to 1318 for the North component, and from 115 to 536 for the vertical component. When compared to the other sensors, these signal-to-noise ratios are relatively large. Regarding the installation of the Rotaphone-CY systems within a maximum distance of 1.2 m (see Figure 2), a much more consistent picture would have been expected. One reason for the strong variation of signal-to-noise ratios can be the incomplete calibration of the Rotaphone-CY systems, which was already mentioned earlier in the text.

### 3.4. Comparison to a Reference Waveform

**Figure 12:**
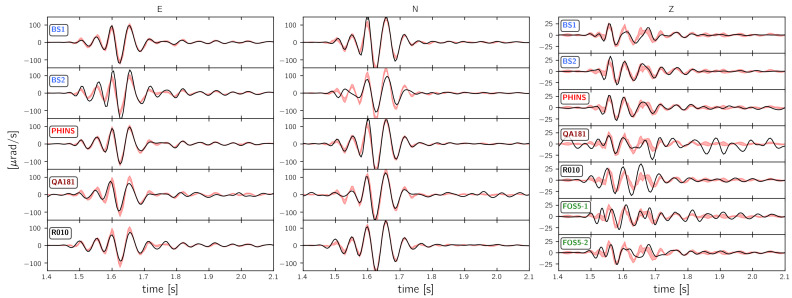
30 Hz low pass filtered waveforms from “expl2” (see Figure 1) recorded by instruments that were placed on top of the monument. The red shaded region depicts the median over all waveform and it has a width of one standard deviation.

Finally, all of the recordings from sensors placed on top of the monument (BS1, BS2, PHINS, QA181, R010, FOS5-1, and FOS5-2) are compared to a hypothetical reference waveform (Figure 12). This reference waveform is computed as the sample-by-sample median waveform among all recordings from rotation sensors mounted on top of the monument. The shaded area shown in Figure 12 represents the range of one standard deviation around this median. This representation summarizes the time domain signal similarity and reveals a very consistent picture for the East and the North component, where only BS2 recorded relatively high amplitudes on the East component and relatively low amplitudes on the North component. The vertical recordings show a different picture: only BS2 and PHINS recordings are most of the time within one standard deviation from the median reference waveform. The other sensors show huge deviations in amplitude as well as in phase.

## 4. Conclusions and Future Work

It is clear that rotation and strain measurements attract more and more interest for geophysical research [17]. Rotation and strain sensing instruments have to be tested and compared extensively in order to establish reliable measurement techniques. The presented experiment brought together more than 40 rotation, strain, and translation sensing instruments with a number of active sources varying in source mechanism, strength, and distance. It provides a unique opportunity not only to compare rotation, strain, and translation sensing techniques among each other, but also to test new ways of data processing, revealing wavefield and source characteristics, as well as source location.

### 4.1. Conclusions

One emerging statement that is derived from this first analysis is that time shifts in the range of several microseconds are not acceptable for this kind of sensors. Data analysis relying on the joint observation of rotation, strain, and translation requires precise amplitude and phase information across the full waveform. This shortcoming originating from either inaccurate time stamping strategies or inappropriate decimation filtering have to be corrected by the manufacturers.

Among the portable and field-deployable broadband rotation rate sensors, the blueSeis-3A sensors have the lowest self-noise levels. In a narrow frequency band from 1 Hz to 20 Hz, the Rotaphone-CY systems show even lower self-noise levels. At this stage and in terms of sensor self-noise, the FARO is the most sensitive portable broadband rotation rate sensor; however, due to its size, only suitable for the use in laboratories or permanent installations. The more compact sensors FOSREM, PHINS, Quadrans, and the MEMS-cube are more suitable for strong motion recordings, for example, in building montoring, active source surveys, and mining activity monitoring.

For the analyzed explosion signal, the **3C broadband rotational seismometers** of the type blueSeis-3A recorded very coherent waveforms looking at a frequency band between 4 Hz and 20 Hz. For instruments that are located in close vicinity to each other, like XB101 and XB102, the coherent part of the spectrum can be extended to even higher frequencies up to 60 Hz. Low pass filtering the recorded waveforms at 30 Hz reveals a more consistent behavior among the individual sensors (Figure 9). However, a relatively wide spread of signal-to-noise ratios and the comparison to a median reference waveform uncovers inconsistencies in the recorded amplitudes that might be partly related to different locations of the sensors and the immature prototype status of single instruments.

At least for the main part of the explosion signal (0.5 s after signal onset) and within a narrow frequency band between 10 Hz and 20 Hz, the **1C broadband rotation rate sensors** of the type FOSREM show coherent recordings among each other as well as compared to vertical recordings of BS1 and BS2 (Figure 6). However, given the close vicinity of the sensors among each other, a more consistent picture would have been expected and the analysis of signal-to-noise ratios and the comparison to the median reference waveform revealed large differences in the amplitude and phase, especially within the later part of the signal.

For the part of the signal where its power clearly exceeds sensor self-noise levels, **strong-motion sensors** show consistent phase locked behavior within a narrow frequency band between 10 Hz and 20 Hz. With relatively high self-noise levels of these sensors, the signal-to-noise ratios vary within an acceptable range and the recorded signals match well with the median reference waveform in terms of phase and amplitude.

The inconsistent coherency spectra between the single **Rotaphone-CY systems** as well as the highly variable signal-to-noise ratios leave open questions that might be resolved as soon as the difficult calibration of the Rotaphone-CY systems can be performed in a suitable laboratory. However, for a narrow frequency band between 10 Hz and 20 Hz, the system R010 shows a coherent waveform in terms of phase and amplitude when compared to other sensors, like the PHINS, QA181, XB100, and BS2, especially for the first 0.5 s after signal onset.

### 4.2. Recommendations for Future Work

This first analysis highlights some important aspects, which have to be taken into account, when testing new instrumentation in the fields of rotation, strain, and translation sensing:

- In order to comply with standards for seismic data recording [60,61], the analog output of a sensor should be recorded with standard seismic recording equipment. In the case of closed loop fiber-optic gyroscopes and distributed acoustic sensing systems, where digital signal processing is required before data can be archived, the data recorder is implemented within the instrument. In this case, recording characteristics such as time keeping accuracy must be accessed in dedicated test procedures. In the presented experiment, we found time shifts between the reference recording of vertical translational acceleration from the station FUR and the transverse rotation rate recordings by maximizing the Pearson cross-correlation coefficient with respect to the applied time shift. Another possibility to access time stamp accuracy and time drifts would be to compare parallel recordings of impulse signals, e.g., generated with a tilt table. In this case, the vertical velocity recording from a reference seismometer-recorder combination should be in phase with the rotation rate recording around the transverse horizontal axis. This method has the advantage of the possibility to reproduce a uniform impulse signal with high accuracy over a long time span of e.g., several days and it can reveal estimates of the recorder time drift with respect to standard seismic reference instruments.
- In a comparative sensor test, all of the instruments under test should experience input motion as identical as possible. Therefore, all the instruments should be co-located as close as possible, being mounted onto a monument that is seismically decoupled from any building structure in order to minimize the local influence of building elements.
- In theory, the transfer function of a fiber-optic gyroscope is flat from DC to the Nyquist frequency. However, the implementation of recording units and closed loop electronics, make it necessary to carefully quantify the frequency response of such a system. Therefore it is desired to develop highly reliable calibration facilities and reference sensors, neither of which is available at the moment.

The frequency content of the signals that were generated during the presented experiment is limited to high frequencies (above 4 Hz). Especially with respect to a broad applicability of ground rotation sensors in fields, such as planetary exploration [62], gravitational wave detection [63], and geodesy [51], extending instrument performance tests and calibrations to low frequencies (below 0.1 Hz) and very week motions (amplitudes smaller than 0.1 rads${}^{-1}$) is necessary. Future test experiments covering an extended frequency range could be realized as huddle tests over a longer time span in the order of several months. This would increase the probability of capturing signals from e.g., regional or teleseismic earthquakes and atmosphere-ground coupling under different atmospheric conditions. Such kinds of experiments should also involve instrument technologies, like cold atom interferometry [64] and beam balances [65]. In addition, the design of special test facilities producing long period weak motions should be considered.

The presented results on basic instrument performance and waveform similarities for explosion signals that were recorded during the huddle test only show a small part of the potential knowledge gain that lies within this unique data set.

## Figures and Tables

**Table 1:** Locations within the seismic bunker, sampling rates, absolute time source and synchronisation method, time shift, and maximum Pearson cross-correlation coefficient (PCC) between station FUR (vertical component) and the evaluated sensors (transverse component). The Global Navigation Satellite System (GNSS) with pulse per second (PPS) output and Network Time Protocol (NTP) were used to retrieve absolute time stamps. The signal-to-noise ratio of the CUBE recording was too low for a reasonable cross-correlation. FARO and ROMY were installed in separate buildings and, therefore, they were not considered to be co-located with the other sensors (n.a. = not applicable).

	Location	Sampling Rate [Hz]	Time Source and Synchronisation Method	Time Shift [s] to FUR	Max PCC
BS1	monument	200	GNSS/PPS ${}^{a}$	−0.005	0.92
BS2	monument	200	GNSS/PPS ${}^{a}$	−0.030	0.92
BGR	floor	200	GNSS/PPS ${}^{a}$	−0.025	0.91
XB100	floor	200	GNSS/PPS ${}^{a}$	−0.030	0.91
XB101	floor	200	GNSS/PPS ${}^{a}$	−0.025	0.93
XB102	floor	200	GNSS/PPS ${}^{a}$	−0.025	0.95
IXBLU	floor	200	GNSS/PPS ${}^{a}$	+0.005	0.91
ISAE	floor	200	GNSS/PPS ${}^{a}$	−0.025	0.93
FOS5-1	monument	1000	NTP	−0.853	0.63
FOS5-2	monument	1000	NTP	−0.896	0.81
FARO	aux. monument	200	GNSS/PPS ${}^{c}$	n.a.	n.a.
ROMY	own building	100	GNSS/PPS ${}^{c}$	n.a.	n.a.
PHINS	monument	200	GNSS/PPS ${}^{b}$	+0.285	0.87
QA181	monument	200	GNSS/PPS ${}^{b}$	+0.230	0.92
QA296	floor	200	GNSS/PPS ${}^{b}$	+0.185	0.87
QA381	floor	200	GNSS/PPS ${}^{b}$	+0.235	0.87
QA384	floor	200	GNSS/PPS ${}^{b}$	+0.195	0.91
CUBE	monument	1000	NTP	n.a.	n.a.
R008	floor	250	GNSS/PPS ${}^{c}$	−0.200	0.75
R010	monument	250	GNSS/PPS ${}^{c}$	−0.200	0.86
R011	floor	250	GNSS/PPS ${}^{c}$	−0.195	0.70
R013	floor	250	GNSS/PPS ${}^{c}$	−0.200	0.86

${}^{a}$ continuously synchronised to PPS signal; ${}^{b}$ free running after initial synchronization to PPS signal; ${}^{c}$ time synchronization done by standard seismic recorder.

## Data Availability

Experiment data are available on request to Felix Bernauer.
